# Dichloridobis(pyrazine-2-carboxamide-κ*N* ^4^)zinc(II)

**DOI:** 10.1107/S1600536812013888

**Published:** 2012-04-04

**Authors:** Sadif A. Shirvan, Sara Haydari Dezfuli

**Affiliations:** a Department of Chemistry, Omidieh Branch, Islamic Azad University, Omidieh, Iran

## Abstract

In the crystal of the title compound, [ZnCl_2_(C_5_H_5_N_3_O)_2_], the mol­ecule has *m* symmetry, with the Zn^II^ cation and Cl^−^ anions located on the mirror plane. The Zn^II^ cation is coordinated by two Cl^−^ anions and two pyrazine-2-carboxamide ligands in a distorted ZnCl_2_N_2_ tetra­hedral geometry. The two pyrazine rings are nearly perpendicular to each other [dihedral angle = 86.61 (10)°]. Inter­molecular N—H⋯O and N—H⋯N hydrogen bonds and weak C—H⋯O inter­actions stabilize the crystal packing.

## Related literature

For related structures, see: Abu-Youssef *et al.* (2006 ▶); Azhdari Tehrani *et al.* (2010 ▶); Goher & Mautner (2000 ▶); Kristiansson (2002 ▶); Mir Mohammad Sadegh *et al.* (2010 ▶); Munakata *et al.* (1997 ▶); Pacigova *et al.* (2008 ▶).
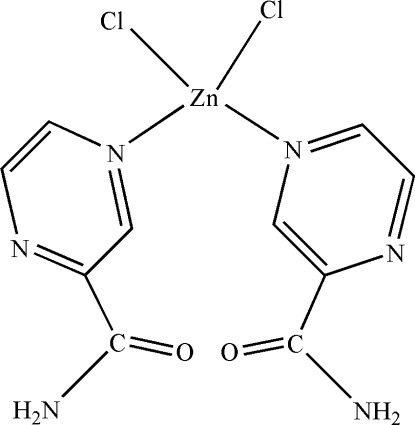


## Experimental

### 

#### Crystal data

[ZnCl_2_(C_5_H_5_N_3_O)_2_]
*M* *_r_* = 382.53
Monoclinic, 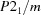

*a* = 5.4296 (5) Å
*b* = 19.7629 (14) Å
*c* = 6.8396 (5) Å
β = 105.131 (7)°
*V* = 708.48 (10) Å^3^
*Z* = 2
Mo *K*α radiation
μ = 2.12 mm^−1^
*T* = 298 K
0.40 × 0.06 × 0.05 mm

#### Data collection

Bruker APEXII CCD area-detector’ diffractometer
Absorption correction: multi-scan (*SADABS*; Bruker, 2001 ▶) *T*_min_ = 0.881, *T*_max_ = 0.902
5777 measured reflections
1441 independent reflections
1064 reflections with *I* > 2σ(*I*)
*R*_int_ = 0.085

#### Refinement

*R*[*F* ^2^ > 2σ(*F* ^2^)] = 0.045
*wR*(*F* ^2^) = 0.093
*S* = 0.97
1441 reflections
100 parameters
H-atom parameters constrained
Δρ_max_ = 0.78 e Å^−3^
Δρ_min_ = −0.65 e Å^−3^

### 

Data collection: *APEX2* (Bruker, 2007 ▶); cell refinement: *SAINT* (Bruker, 2007 ▶); data reduction: *SAINT*; program(s) used to solve structure: *SHELXTL* (Sheldrick, 2008 ▶); program(s) used to refine structure: *SHELXTL*; molecular graphics: *SHELXTL*; software used to prepare material for publication: *SHELXTL*.

## Supplementary Material

Crystal structure: contains datablock(s) I, global. DOI: 10.1107/S1600536812013888/xu5500sup1.cif

Structure factors: contains datablock(s) I. DOI: 10.1107/S1600536812013888/xu5500Isup2.hkl

Additional supplementary materials:  crystallographic information; 3D view; checkCIF report

## Figures and Tables

**Table 1:** Selected bond lengths (Å)

Zn1—N1	2.085 (3)
Zn1—Cl1	2.1945 (16)
Zn1—Cl2	2.1888 (16)

**Table 2:** Hydrogen-bond geometry (Å, °)

*D*—H⋯*A*	*D*—H	H⋯*A*	*D*⋯*A*	*D*—H⋯*A*
N3—H3*B*⋯O1^i^	0.86	2.02	2.875 (5)	175
N3—H3*C*⋯N2^ii^	0.86	2.61	3.205 (5)	128
C3—H3⋯O1^iii^	0.93	2.44	3.357 (5)	170

Symmetry codes: (i) 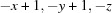; (ii) 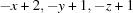; (iii) 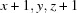.

**Fig. 1.**
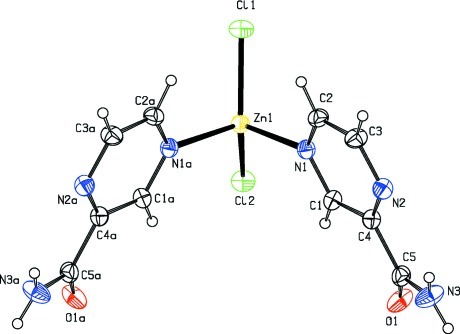
The molecular structure of the title molecule, with the atom-numbering scheme. Displacement ellipsoids are drawn at the 30% probability level. [Symmetry codes: (a) x,1/2 - y,z].

**Fig. 2.**
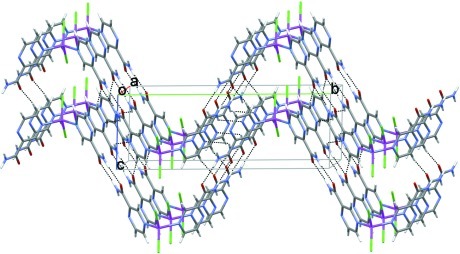
Unit-cell packing diagram for title molecule. Hydrogen bonds are shown as dashed lines

## supplementary crystallographic information

### Comment

Pyrazine-2-carboxamide (pzc), is a good ligand, and a few complexes with pzc have been prepared, such as that of mercury (Azhdari Tehrani *et al.*, 2010; Mir Mohammad Sadegh *et al.*, 2010), vanadium (Pacigova *et al.*, 2008), manganese (Abu-Youssef *et al.*, 2006) and copper (Kristiansson, 2002; Munakata *et al.*, 1997; Goher & Mautner, 2000). Here, we report the synthesis and structure of the title compound.

The asymmetric unit of the title compound, (Fig. 1), contains one Zn^II^ atom, two Cl atoms and one pyrazine-2-carboxamide ligand. The Zn^II^ atom is four-coordinated in a distorted tetrahedral configuration by two N atoms from two pyrazine-2-carboxamide ligands and two terminal Cl atoms. The Zn—Cl and Zn—N bond lengths and angles are collected in Table 1.

In the crystal structure, intermolecular N—H···O, N—H···N and C—H···O hydrogen bonds (Table 2, Fig. 2) may stabilize the structure.

### Experimental

A solution of pyrazine-2-carboxamide (0.25 g, 2.0 mmol) in methanol (10 ml) was added to a solution of ZnCl_2_ (0.13 g, 1.0 mmol) in methanol (10 ml) and the resulting colorless solution was stirred for 15 min at room temperature. This solution was left to evaporate slowly at room temperature. After one week, colorless plate crystals of the title compound were isolated (yield 0.30 g, 78.4%).

### Refinement

All H atoms were positioned geometrically, with C—H = 0.93 and N—H = 0.86 Å, and constrained to ride on their parent atoms with U_iso_(H) = 1.2U_eq_(C,N).

### Crystal data

**Table:**

[ZnCl_2_(C_5_H_5_N_3_O)_2_]	*F*(000) = 384
*M**_r_* = 382.53	*D*_x_ = 1.793 Mg m^−^^3^
Monoclinic, *P*2_1_/*m*	Mo *K*α radiation, λ = 0.71073 Å
Hall symbol: -P 2yb	Cell parameters from 5777 reflections
*a* = 5.4296 (5) Å	θ = 2.1–26.0°
*b* = 19.7629 (14) Å	µ = 2.12 mm^−^^1^
*c* = 6.8396 (5) Å	*T* = 298 K
β = 105.131 (7)°	Plate, colorless
*V* = 708.48 (10) Å^3^	0.40 × 0.06 × 0.05 mm
*Z* = 2	

### Data collection

**Table:**

Bruker APEXII CCD area-detector' diffractometer	1441 independent reflections
Radiation source: fine-focus sealed tube	1064 reflections with *I* > 2σ(*I*)
Graphite monochromator	*R*_int_ = 0.085
ω scans	θ_max_ = 26.0°, θ_min_ = 2.1°
Absorption correction: multi-scan (*SADABS*; Bruker, 2001)	*h* = −6→6
*T*_min_ = 0.881, *T*_max_ = 0.902	*k* = −21→24
5777 measured reflections	*l* = −8→8

### Refinement

**Table:**

Refinement on *F*^2^	Primary atom site location: structure-invariant direct methods
Least-squares matrix: full	Secondary atom site location: difference Fourier map
*R*[*F*^2^ > 2σ(*F*^2^)] = 0.045	Hydrogen site location: inferred from neighbouring sites
*wR*(*F*^2^) = 0.093	H-atom parameters constrained
*S* = 0.97	*w* = 1/[σ^2^(*F*_o_^2^) + (0.0467*P*)^2^] where *P* = (*F*_o_^2^ + 2*F*_c_^2^)/3
1441 reflections	(Δ/σ)_max_ = 0.003
100 parameters	Δρ_max_ = 0.78 e Å^−^^3^
0 restraints	Δρ_min_ = −0.65 e Å^−^^3^

### Special details

**Table:**

Geometry. All e.s.d.'s (except the e.s.d. in the dihedral angle between two l.s. planes) are estimated using the full covariance matrix. The cell e.s.d.'s are taken into account individually in the estimation of e.s.d.'s in distances, angles and torsion angles; correlations between e.s.d.'s in cell parameters are only used when they are defined by crystal symmetry. An approximate (isotropic) treatment of cell e.s.d.'s is used for estimating e.s.d.'s involving l.s. planes.
Refinement. Refinement of *F*^2^ against ALL reflections. The weighted *R*-factor *wR* and goodness of fit *S* are based on *F*^2^, conventional *R*-factors *R* are based on *F*, with *F* set to zero for negative *F*^2^. The threshold expression of *F*^2^ > σ(*F*^2^) is used only for calculating *R*-factors(gt) *etc*. and is not relevant to the choice of reflections for refinement. *R*-factors based on *F*^2^ are statistically about twice as large as those based on *F*, and *R*- factors based on ALL data will be even larger.

### Fractional atomic coordinates and isotropic or equivalent isotropic displacement parameters (Å^2^)

**Table:**

	*x*	*y*	*z*	*U*_iso_*/*U*_eq_	
C1	0.3963 (7)	0.3616 (2)	0.4810 (6)	0.0307 (9)	
H1	0.2583	0.3512	0.3724	0.037*	
C2	0.6226 (8)	0.3464 (2)	0.8085 (6)	0.0364 (10)	
H2	0.6435	0.3262	0.9347	0.044*	
C3	0.8016 (8)	0.3930 (2)	0.7802 (6)	0.0391 (10)	
H3	0.9428	0.4022	0.8873	0.047*	
C4	0.5722 (7)	0.4094 (2)	0.4563 (6)	0.0310 (8)	
C5	0.5370 (8)	0.4445 (2)	0.2579 (6)	0.0366 (9)	
N1	0.4213 (6)	0.33021 (16)	0.6574 (5)	0.0312 (7)	
N2	0.7776 (6)	0.42473 (18)	0.6048 (5)	0.0366 (8)	
N3	0.7336 (7)	0.4801 (2)	0.2331 (6)	0.0535 (11)	
H3B	0.7210	0.5022	0.1226	0.064*	
H3C	0.8736	0.4812	0.3276	0.064*	
O1	0.3309 (6)	0.44069 (17)	0.1293 (4)	0.0506 (8)	
Zn1	0.18582 (12)	0.2500	0.69002 (10)	0.0304 (2)	
Cl1	0.1855 (3)	0.2500	1.0108 (2)	0.0480 (4)	
Cl2	−0.1427 (3)	0.2500	0.4244 (2)	0.0416 (4)	

### Atomic displacement parameters (Å^2^)

**Table:**

	*U*^11^	*U*^22^	*U*^33^	*U*^12^	*U*^13^	*U*^23^
C1	0.0264 (19)	0.030 (2)	0.034 (2)	−0.0008 (16)	0.0050 (17)	0.0030 (17)
C2	0.040 (2)	0.038 (2)	0.028 (2)	−0.0004 (18)	0.0041 (18)	0.0030 (18)
C3	0.036 (2)	0.046 (3)	0.030 (2)	−0.0092 (19)	−0.0004 (18)	0.0013 (19)
C4	0.0278 (19)	0.030 (2)	0.034 (2)	−0.0010 (16)	0.0063 (16)	−0.0002 (17)
C5	0.041 (2)	0.031 (2)	0.036 (2)	−0.0065 (18)	0.0050 (19)	0.0002 (18)
N1	0.0300 (16)	0.0295 (18)	0.0341 (19)	−0.0027 (14)	0.0081 (14)	−0.0011 (14)
N2	0.0325 (18)	0.037 (2)	0.037 (2)	−0.0053 (14)	0.0032 (15)	−0.0011 (15)
N3	0.041 (2)	0.073 (3)	0.042 (2)	−0.0229 (19)	0.0026 (17)	0.017 (2)
O1	0.0432 (17)	0.058 (2)	0.0413 (18)	−0.0198 (15)	−0.0053 (15)	0.0173 (16)
Zn1	0.0290 (3)	0.0313 (4)	0.0329 (4)	0.000	0.0114 (3)	0.000
Cl1	0.0570 (10)	0.0575 (11)	0.0328 (8)	0.000	0.0174 (7)	0.000
Cl2	0.0303 (7)	0.0525 (10)	0.0408 (8)	0.000	0.0069 (6)	0.000

### Geometric parameters (Å, º)

**Table:**

C1—N1	1.332 (5)	C5—O1	1.232 (5)
C1—C4	1.384 (5)	C5—N3	1.326 (5)
C1—H1	0.9300	N1—Zn1	2.085 (3)
C2—N1	1.333 (5)	N3—H3B	0.8600
C2—C3	1.388 (6)	N3—H3C	0.8600
C2—H2	0.9300	Zn1—N1	2.085 (3)
C3—N2	1.330 (5)	Zn1—N1^i^	2.085 (3)
C3—H3	0.9300	Zn1—Cl1	2.1945 (16)
C4—N2	1.333 (5)	Zn1—Cl2	2.1888 (16)
C4—C5	1.491 (6)		
			
N1—C1—C4	121.2 (4)	N3—C5—C4	116.6 (4)
N1—C1—H1	119.4	C1—N1—C2	117.4 (3)
C4—C1—H1	119.4	C1—N1—Zn1	122.1 (3)
N1—C2—C3	120.9 (4)	C2—N1—Zn1	120.1 (3)
N1—C2—H2	119.6	C3—N2—C4	116.4 (3)
C3—C2—H2	119.6	C5—N3—H3B	120.0
N2—C3—C2	122.1 (4)	C5—N3—H3C	120.0
N2—C3—H3	118.9	H3B—N3—H3C	120.0
C2—C3—H3	118.9	N1—Zn1—N1^i^	99.00 (18)
N2—C4—C1	121.9 (4)	N1—Zn1—Cl2	107.51 (10)
N2—C4—C5	118.1 (3)	N1^i^—Zn1—Cl2	107.51 (10)
C1—C4—C5	120.0 (3)	N1—Zn1—Cl1	105.53 (9)
O1—C5—N3	123.5 (4)	N1^i^—Zn1—Cl1	105.53 (9)
O1—C5—C4	119.9 (4)	Cl2—Zn1—Cl1	128.07 (6)
			
N1—C2—C3—N2	1.9 (7)	C3—C2—N1—Zn1	170.7 (3)
N1—C1—C4—N2	2.1 (6)	C2—C3—N2—C4	−0.2 (6)
N1—C1—C4—C5	−179.0 (4)	C1—C4—N2—C3	−1.7 (6)
N2—C4—C5—O1	−167.5 (4)	C5—C4—N2—C3	179.4 (4)
C1—C4—C5—O1	13.5 (6)	C1—N1—Zn1—N1^i^	96.5 (3)
N2—C4—C5—N3	10.8 (6)	C2—N1—Zn1—N1^i^	−75.5 (3)
C1—C4—C5—N3	−168.2 (4)	C1—N1—Zn1—Cl2	−15.2 (3)
C4—C1—N1—C2	−0.3 (6)	C2—N1—Zn1—Cl2	172.9 (3)
C4—C1—N1—Zn1	−172.5 (3)	C1—N1—Zn1—Cl1	−154.6 (3)
C3—C2—N1—C1	−1.5 (6)	C2—N1—Zn1—Cl1	33.5 (3)

Symmetry code: (i) *x*, −*y*+1/2, *z*.

### Hydrogen-bond geometry (Å, º)

**Table:**

*D*—H···*A*	*D*—H	H···*A*	*D*···*A*	*D*—H···*A*
N3—H3*B*···O1^ii^	0.86	2.02	2.875 (5)	175
N3—H3*C*···N2^iii^	0.86	2.61	3.205 (5)	128
C3—H3···O1^iv^	0.93	2.44	3.357 (5)	170

Symmetry codes: (ii) −*x*+1, −*y*+1, −*z*; (iii) −*x*+2, −*y*+1, −*z*+1; (iv) *x*+1, *y*, *z*+1.
